# A Scoping Review of Clinical Trials on the Efficacy of Curcumin and Its Formulations for Wound Healing

**DOI:** 10.1111/jocd.70615

**Published:** 2025-12-13

**Authors:** Mohammad Mahdi Parvizi, Ali Arefkia, Yasamin Dehghan, Mohammad Ali Salimi, Negin Fazelzadeh Haghighi

**Affiliations:** ^1^ Molecular Dermatology Research Center Shiraz University of Medical Sciences Shiraz Iran; ^2^ Research Center for Traditional Medicine and History of Medicine Shiraz University of Medical Sciences Shiraz Iran; ^3^ Department of Medical Journalism, School of Paramedical Sciences Shiraz University of Medical Sciences Shiraz Iran; ^4^ Student Research Committee, School of Medicine Shiraz University of Medical Sciences Shiraz Iran; ^5^ Department of Dermatology, School of Medicine Shiraz University of Medical Sciences Shiraz Iran; ^6^ Department of Pathology, School of Medicine Shiraz University of Medical Sciences Shiraz Iran

**Keywords:** complementary therapies, curcumin, Persian medicine, plant extracts, wound healing

## Abstract

**Background:**

Curcumin, the main component of turmeric, may be effective in wound healing due to its anti‐inflammatory and antibacterial effects. Although pre‐clinical studies are promising, a comprehensive clinical review is lacking. This scoping review examines clinical trials on the efficacy of curcumin in wound healing.

**Method:**

This study was conducted in accordance with the PRISMA‐ScR guidelines and involved a comprehensive search of PubMed, Scopus, Web of Science, and Cochrane Library databases for clinical trials on various forms of curcumin for wound healing in humans, from January 1, 2000 to May 31, 2025.

**Results:**

A total of 920 results were retrieved through the search, of which 19 clinical trials met the predefined inclusion and exclusion criteria and were included in our study. Most studies (14 out of 19) were randomized controlled trials. Curcumin was used in various dosages and treatment durations, and in multiple forms, including topical and oral formulations. Curcumin improved wound healing compared to placebo or conventional care in 89% of the studies. Additionally, no adverse events were reported in 84% of the studies, with minor and temporary side effects observed in the remaining cases.

**Conclusions:**

According to the findings of the current study, curcumin is a safe and effective adjuvant for improving wound healing. However, the significant heterogeneity observed among clinical trials limits the ability to develop consistent treatment guidelines. Future studies should focus on large‐scale, standardized trials to better define the role of curcumin in wound care.

## 1 Introduction

Human skin is the body's first line of defense against environmental stresses and also plays an essential role in maintaining physiological homeostasis and immunity [1]. A wound, a disruption of this vital barrier, can be classified as acute, resolving within a few weeks, or chronic, in which the healing process may persist for months due to persistent inflammation [2, 3, 4]. Chronic wounds have become a major public health concern in the past decades due to their high prevalence, primarily resulting from an aging population and the significant rise in obesity and systemic disorders such as diabetes, heart failure, and peripheral artery disease [5, 6]. Recent studies indicate that 1%–2% of individuals suffer from chronic wounds during their life time [7]. This issue imposes a substantial clinical and financial burden on healthcare systems, with annual treatment costs in the United States reaching tens of billions of dollars. It also highlights the urgent need for novel and efficient therapies for chronic wound [8, 9].

There are four stages in the normal biological process of wound healing: hemostasis, inflammation, proliferation, and remodeling [10]. The initial phase, hemostasis, involves the formation of fibrin clots and platelet aggregates, leading to the cessation of blood flow [11]. The inflammatory phase is characterized by the removal of debris by immune cells, creating a complex network of growth factors and cytokines, including Transforming Growth Factor beta (TGF‐β), Fibroblast Growth Factor (FGF), and Tumor Necrosis Factor alpha (TNF‐α), that signal the initiation of the tissue healing process [12]. In the subsequent proliferative phase, angiogenesis, formation of a collagen type III matrix, and re‐epithelialization occur. Finally, the remodeling phase involves the maturation of this newly formed tissue, restoring the skin's functional integrity by gradually replacing collagen type III with the more durable collagen type I. Failure to progress smoothly through this cascade can result in a chronic, non‐healing wound [11, 13].

Various treatments have been used for chronic wounds. As medicinal plants are considered more readily available, less expensive, and less likely to cause adverse side effects than synthetic medications, they have long been employed by many cultures, particularly in Asia and Africa [14, 15, 16]. Curcumin, the principal active component in *Curcuma longa* (turmeric), is particularly notable among these agents. This compound, used for medicinal purposes for over 6000 years, may be considered an effective therapy for wound healing [17, 18]. Given its well‐established antibacterial, antioxidant, and anti‐inflammatory properties, curcumin can modulate the wound environment through all stages of healing [19]. Specifically, it supports tissue remodeling by enhancing TGF‐β synthesis, stimulates the proliferative phase through fibroblast migration and collagen deposition, and regulates inflammation by down‐regulating important cytokines including interleukin 1 (IL‐1) and TNF‐α [20, 21].

While some studies have previously evaluated the benefits of curcumin [22, 23], a comprehensive analysis is still required to elucidate its various therapeutic applications in wound healing. The main objective of this study is to map the scope, diversity, and nature of the available data. As clinical trials provide the most direct evidence for the therapeutic use of drugs in humans, this review primarily focuses on such studies. In this study, we aim to identify and classify existing clinical trial literature to summarize key features, including the types of wounds examined, the range of curcumin formulations and dosages applied, the outcome measures assessed, and the reported safety data. Ultimately, we provide a comprehensive overview of the current state of clinical research, highlighting existing knowledge gaps, and recommending directions for future studies.

## 2 Method

This study aimed to investigate the efficacy of curcumin and its derivatives in treating chronic wounds. We performed this scoping review in accordance with the Preferred Reporting Items for Systematic Reviews and Meta‐Analyses extension for Scoping Reviews (PRISMA‐ScR) standards and the methodological framework for scoping reviews set out by Arksey and O'Malley [24].

### 2.1 Research Question

Using the Population, Intervention, Comparison, and Outcome (PICO) structure, the following research questions were developed for this review:

- *Population*: Individuals with cutaneous wounds of any kind, including surgical, traumatic, chronic, infectious, and burn‐related wounds, regardless of age.
- *Intervention*: Any treatment that involves the topical or systemic administration of curcumin or its compounds, such as turmeric or curcuminoids.
- *Comparison*: The control or comparison group in the clinical studies that were included may have received no therapy, a standard course of treatment, or a placebo.
- *Outcome*: Metrics related to wound healing, including healing rate, duration to complete closure, and qualitative or quantitative enhancements in wound characteristics, were the focus. Pain reduction and adverse events were secondary outcomes.

### 2.2 Eligibility Criteria

We searched for all types of clinical trials, including pilot studies, non‐randomized trials, and randomized controlled trials (RCTs) from January 1, 2000 to May 31, 2025. All included studies were published in the English language. In terms of content, our study included research on the application of substances derived from turmeric or curcumin for the healing of wounds of any kind. Duplicate publications, studies in which the primary intervention was not curcumin‐based, studies other than wound healing as a primary or secondary result, in vitro or animal studies, review articles, editorials, letters, and case reports were excluded from the present study.

### 2.3 Information Sources and Search Strategy

The following electronic databases were searched extensively: the Cochrane Library, Web of Science, PubMed, and Scopus. Google Scholar was also used to find further relevant articles through both forward and backward citation searches. The search was conducted until May 2025, without any restrictions on time or date. The search approach used the Boolean operators “AND” and “OR” to combine the keywords and MeSH phrases associated with the intervention and the outcome. The main search terms were: (“Clinical Trial” OR “clinical study” OR “randomized controlled trial” OR “RCT” OR “intervention study”) **AND** (“Curcumin” OR “turmeric” OR “ *Curcuma longa* ” OR “diferuloylmethane” OR “curcuminoids”) **AND** (“Wound Healing” OR “wound repair” OR “tissue regeneration” OR “skin healing” OR “wound closure” OR “dermal healing”).

### 2.4 Study Selection

The research selection process involved multiple steps. Initially, all identified citations were imported into a reference management system, and duplicate citations were removed. Subsequently, two authors, M.M.P. and A.A., independently screened the titles and abstracts of the studies to assess their relevance based on predefined eligibility criteria. Any disagreements about inclusion were resolved by consensus or, if necessary, by consulting N.F.H.

### 2.5 Data Charting and Synthesis of Results

In the data charting process, M.M.P., Y.D., N.F.H., and M.A.S. participated in reviewing the selected articles, extracting data, and confirming their accuracy. Subsequently, M.M.P., Y.D., and A.A. organized the extracted data using a researcher‐made tabulated form. Based on the indicated results, the findings were categorized and analyzed according to the curcumin formulation and wound type. A quantitative meta‐analysis was not conducted.

### 2.6 Data Items

The extracted data included Author(s) and year of publication; details about the type of clinical trial and sample size; participants' characteristics, formulations, dosage, and route of administration; frequency and duration of treatment; details of the control group intervention; outcomes and methods of assessment; study results; and any reported side effects or complications.

### 2.7 Ethics Statement

This study was approved by the Research Ethics Committee of Shiraz University of Medical Sciences (Ethics Code: IR.SUMS.MED.REC.1404.036).

## 3 Results

### 3.1 Study Selection

**FIGURE 1:**
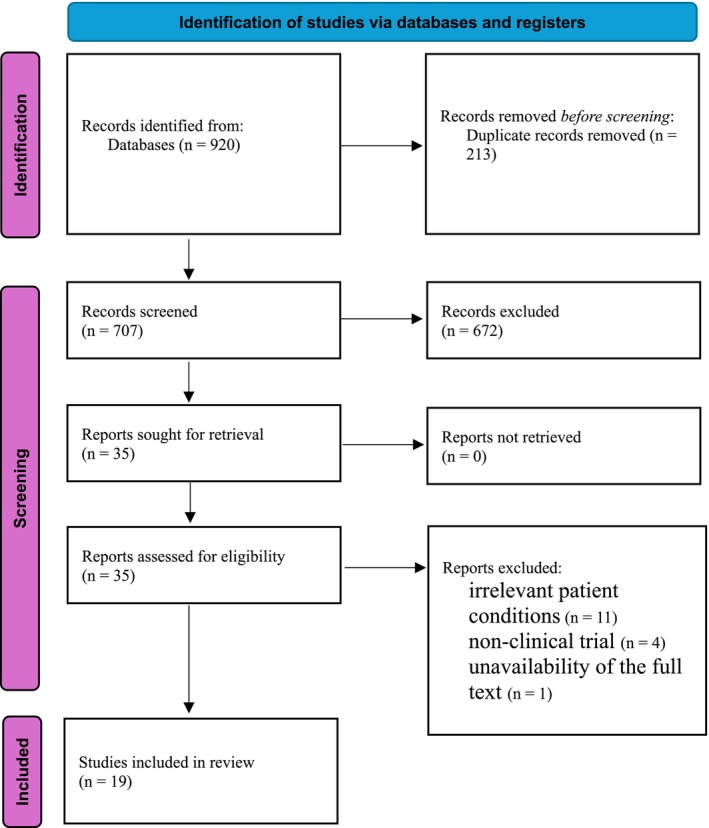
PRISMA‐ScR diagram of the study.

The systematic search and study selection process is detailed in a PRISMA‐ScR flow diagram (Figure 1). After the initial thorough examination of the selected databases, 920 results were identified. Following the removal of 213 duplicates, 707 unique articles underwent title and abstract screening. At this stage, 672 studies were excluded, mainly because they were non‐clinical review articles, or those that addressed topics unrelated to wound healing. This screening yielded 35 articles for full‐text review. A careful review of the full‐text publications led to the exclusion of 16 additional studies due to inappropriate patient conditions (*n* = 11), non‐clinical trial designs (*n* = 4), or the absence of full text (*n* = 1). Finally, 19 clinical studies met the eligibility criteria and were included in the data extraction and narrative synthesis.

### 3.2 Characteristics of Included Studies

Over the last two decades, there has been an increasing interest in the clinical application of curcumin for wound treatment, as evidenced by the 19 papers included in this review (Table 1). The research designs were mainly randomized controlled trials (*n* = 14) [25, 26, 27, 28, 29, 30, 31, 32, 33, 34, 35, 39, 40, 41, 42], with three pilot trials [36, 37, 43] and one quasi‐experimental study (*n* = 1) [38]. The sample sizes varied greatly, ranging from 11 to 178 participants. Demographic characteristics were diverse, ranging from 17 to 85 years old and included different types of wounds. While thirteen studies had both male and female participants [25, 30, 31, 32, 34, 35, 36, 37, 39, 40, 41, 42, 43] a subgroup targeted gender‐specific conditions, with five trials including only females for post‐partum wounds (e.g., episiotomy) [26, 27, 29, 33, 38] and one trial containing only males for chronic skin lesions caused by mustard gas exposure [28].

**TABLE 1:** Summary of the included clinical trials on curcumin for wound healing.

Author(s), year	Study design	F/M	Age, mean (SD)	Age range	Wound type	Intervention	Control group(s)	Key efficacy outcomes	Reported adverse events
Chainani‐Wu et al. (2007) [25]	RCT, placebo‐controlled	23/10	60.6 (8.68)	> 21 years	Oral Lichen Planus	Oral curcuminoid capsules (2 g/day)	Placebo	No significant difference in symptom reduction; study terminated for futility	Well tolerated; mild, non‐significant GI issues
Golmakani et al. (2008) [26]	RCT, double‐blind	63/0	—	17–35 years	Episiotomy	Topical 5% turmeric ointment	Placebo (vaseline)	Significant reduction in REEDA score and faster healing time (10 vs. 14 days)	None reported
Esmaeili Vardanjani et al. (2012) [27]	RCT, double‐blind	117/0	23.46 (4.81)	—	Episiotomy	Topical curcumin solution	Povidone‐iodine solution	Significant improvement in REEDA score and pain reduction at day 10	None reported
Panahi et al. (2012) [28]	RCT, placebo‐controlled	0/80	—	37–59	Mustard gas‐induced skin lesions	Oral curcumin capsules (1 g/day)	Placebo	Significant reduction in itch, inflammatory markers (IL‐8, hs‐CRP), and improved quality of life	Mild GI issues in 3 patients
Mahmudi et al. (2015) [29]	RCT, triple‐arm	162/0	26.45 (5.74)	—	Cesarean section wound	Topical turmeric cream	Placebo/No treatment	Significant reduction in REEDA scores compared to controls	None reported
Anuradha et al. (2015) [30]	RCT (Split‐Mouth)	30 (total)	—	25–60	Chronic Periodontitis	Topical curcumin gel adjunct to SRP	SRP alone	Significant reduction in Plaque Index, Gingival Index, and Probing Depth	None reported
Lone et al. (2017) [31]	RCT	178 (total)	—	—	Alveolar Osteitis (Dry Socket)	Topical turmeric and mustard oil dressing	Zinc Oxide Eugenol (ZOE) dressing	Faster wound healing and significant reduction in pain and inflammation compared to ZOE	None reported
Chaitanya et al. (2019) [32]	RCT	49/26	51.85 (13.14)	—	Chemoradiation‐induced Oral Mucositis	Topical zinc formula with curcumin	Zinc alone/Standard care	Reduced severity of oral mucositis compared to controls	Not specified
Nikpour et al. (2019) [33]	RCT, triple‐arm	89/0	24.66 (3.94)	—	Episiotomy	Topical 2% curcumin cream	Honey cream/Placebo	No significant difference in pain relief or wound healing scores between groups	No significant irritation reported
Shah et al. (2020) [34]	Pilot RCT, triple‐blind	22/52	54.34 (13.78)	26–96	Radiation‐induced Oral Mucositis (RIOM)	0.1% curcumin mouthwash	0.15% Benzydamine mouthwash	Significantly delayed onset of RIOM compared to benzydamine	Mild burning sensation (1 patient per group)
Mokhtari et al. (2020) [35]	RCT, placebo‐controlled	11/39	56.6 (10.55)	—	Diabetic Foot Ulcer (DFU)	Oral 80 mg nanocurcumin capsules	Placebo	Significant improvement in metabolic markers (glucose, insulin); no significant change in wound size	None reported
Mugilan et al. (2020) [36]	Pilot RCT	11 (total)	57.64	46–63	Extraction socket (diabetic patients)	Topical curcumin gel	Standard care	Significant reduction in socket width and postoperative pain	None reported
Raman et al. (2020) [37]	Pilot RCT	41/19	24.67	—	Recurrent Aphthous Ulcer	Topical 2% curcumin gel	0.1% Triamcinolone paste	Comparable efficacy to triamcinolone in reducing ulcer size and pain	Mild cooling sensation
Mutia et al. (2021) [38]	Quasi‐experimental	45/0	—	≤ 35	Perineal wound	Topical 5% & 10% turmeric extract solution	Standard drugs	Significantly faster healing based on REEDA scores compared to standard care	None reported
Bakhshi et al. (2022) [39]	RCT, double‐blind	48 (total)	—	—	Recurrent Aphthous Stomatitis	1% curcumin nanomicelle gel	2% curcumin gel	Significant reduction in pain and lesion size in both groups, suggesting efficacy of both formulations	None reported
Pandya et al. (2023) [40]	RCT (Split‐Mouth)	13/8	56	—	Extraction socket	Topical curcumin gel	Placebo gel	Significantly higher healing scores and lower pain scores	None reported
Sanpinit et al. (2024) [41]	RCT	50 (total)	—	—	Diabetic Foot Ulcer (DFU)	Topical polyherbal solution (with curcumin)	Standard care	Significantly higher rate of complete wound closure (76% vs. 16% in control)	None reported
Hessari et al. (2024) [42]	RCT (Split‐Mouth)	34 (total)	—	—	Extraction site preservation	Nanocurcumin spongy membrane	No treatment	Significant improvement in soft tissue (gum) thickness; no significant effect on bone repair	None reported
Gomes TFS et al. (2025) [43]	Pilot controlled trial	3/12	59	18–75	Diabetic Foot Ulcers (DFUs)	Topical natural latex‐based biomembrane + curcumin‐loaded liposomes + red LED phototherapy	Standard care, Natural latex biomembrane + LED phototherapy	Statistically significant wound healing progression, with 89.8% mean wound closure by day 45, superior to both control groups	None reported

### 3.3 Intervention Characteristics

The trials comprised a wide range of curcumin‐based therapies, with varying formulations, delivery routes, dosages, and durations.

#### 3.3.1 Formulations and Administration Routes

The treatments were administered through both topical and systemic methods. Topical administration was the most prevalent method, used in sixteen trials with formulations such as gels, ointments, creams, solutions, medicated dressings, and bio‐membranes [26, 27, 29, 30, 31, 32, 33, 34, 36, 37, 38, 39, 40, 41, 42, 43]. Three studies investigated the systemic effects of oral curcumin, usually in the form of capsules [25, 28, 35]. Additionally, one study investigated the synergistic effects of mixing curcumin with other medicinal substances, specifically zinc, in topical formulations [32].

#### 3.3.2 Dosage, Duration, and Comparators

The dosage regimes and treatment durations varied. Topical preparations typically contained curcumin doses ranging from 1% to 10%, applied two to three times daily. The duration of the interventions varied depending on the wound type and study protocol, ranging from a five‐day application to a 12‐week treatment course for chronic diseases.

The comparison groups were diverse: Eight trials used a placebo control [25, 26, 28, 29, 35, 40], allowing direct assessment of curcumin efficacy; Six studies compared curcumin to standard active treatments, including antiseptics such as povidone‐iodine [27] and anti‐inflammatory medications like benzydamine [34] or triamcinolone acetonide [37]. The remaining studies either employed no treatment [30, 42], alternative options such as basic gel [31, 33, 39, 41], or routine hospital wound care as the control [32, 36, 38, 43].

### 3.4 Efficacy and Clinical Outcomes

The vast majority of the trials (16 of 19, or 84%) showed significantly improved outcomes across different types of wounds, including surgical wounds, oral lesions, and chronic wounds. The key details are as follows:

- Surgical and Post‐Procedural Wounds: Research has consistently demonstrated significant benefits for acute surgical wounds, including episiotomies, cesarean sections, and perineal repairs. The findings from five trials indicated that the use of topical curcumin resulted in a quicker time to complete wound closure and statistically significant reductions in REEDA (Redness, Edema, Ecchymosis, Discharge, Approximation) scores compared to the control groups [26, 27, 29, 31, 38].
- Oral and Dental Lesions: Curcumin has demonstrated significant effectiveness in treating various oral and dental conditions. It notably reduces pain and inflammation, accelerates healing of dental extraction sites, delays the onset of severe radiation‐induced oral mucositis, and decreases the size and discomfort of recurrent aphthous ulcers, as indicated by eight out of ten studies in this area [30, 31, 32, 34, 36, 37, 39, 40]. Several trials found that curcumin was as effective as the standard corticosteroid treatment, triamcinolone. However, one trial on oral lichen planus reported no significant difference between curcumin and placebo [25].
- Chronic Wounds and Other Lesions: Three long‐term studies on diabetic foot ulcers showed promising results. In one study, individuals taking oral curcumin demonstrated significant improvements in metabolic indicators, including insulin resistance and fasting blood glucose levels [35]. Another study found that 76% of the patients achieved complete wound closure using a topical polyherbal preparation containing curcumin. Furthermore, oral curcumin significantly reduced the severity of itching and lowered the key inflammatory biomarkers in a trial involving persistent skin lesions caused by mustard gas exposure [41]. Reinforcing these findings, a pilot study on diabetic foot ulcers applied a novel topical therapy combining a curcumin‐loaded biomembrane with LED phototherapy. This advanced dressing resulted in a statistically significant mean wound closure of 89.8% over 45 days [43].

It is important to note that, in two trials, curcumin did not demonstrate a statistically significant therapeutic advantage over placebo or control interventions, despite generally encouraging results [25, 33].

### 3.5 Safety and Tolerability

All reviewed studies indicated that curcumin had an excellent safety profile, with 84% (16 of 19 studies) reporting no adverse events related to the intervention [25, 26, 27, 29, 30, 31, 32, 33, 35, 36, 38, 39, 40, 41, 42, 43]. In the four studies that reported side effects, these were consistently minor and temporary. Mild gastrointestinal discomfort, such as bloating, diarrhea, or nausea, was common in oral administration [25, 28]. Additionally, sporadic sensations of burning or cooling were reported with topical applications [34, 37]. Overall, curcumin is well‐tolerated and has a good safety profile for both systemic and topical use in wound care settings, according to this body of research.

## 4 Discussion

In this scoping review, we analyzed and collected data from 19 clinical trials which evaluated the therapeutic potential of curcumin for wound healing. The primary finding is that curcumin, in both topical and systemic formulations, is a safe and effective adjuvant for the treatment of a wide range of wounds. Most of the studies included in the review (89%) reported statistically significant positive outcomes, such as a faster healing period, reduced inflammation and pain, and improved clinical outcomes for surgical, postoperative, and chronic wounds. Additionally, the evidence suggests that curcumin has a high safety profile; most trials reported no adverse effects, and all the reported side effects were minor and temporary.

Curcumin targets several key processes in wound healing, including inflammation reduction, fibroblast migration, and collagen synthesis [44]. This provides compelling evidence for the consistent effectiveness observed in the trials conducted. Curcumin reduces pro‐inflammatory cytokines, such as TNF‐α, IL‐1, and inhibits the Nuclear Factor kappa‐light‐ chain‐enhancer of activated B‐cells (NF‐κB) signaling pathway. This action is associated with documented decreases in clinical indicators of inflammation, including REEDA scores [38]. Curcumin facilitates the healing process by shortening the inflammatory phase [14, 19]. It also enhances collagen synthesis in the proliferative phase by stimulating the migration of fibroblasts to the wound site. Moreover, Curcumin promotes angiogenesis by upregulating Vascular Endothelial Growth Factor (VEGF), which induces wound closure, as observed in multiple trials [45, 46]. Similarly, natural compounds such as docosahexaenoic acid (DHA) and eicosapentaenoic acid (EPA) have demonstrated modulatory effects on apoptotic regulators' potential to target apoptotic pathways in tissue repair and inflammation control [47]. This further supports the idea that bioactive natural agents can exert cytoprotective and anti‐inflammatory actions through the regulation of molecular mechanisms involved in apoptosis and oxidative stress. Its high antioxidant properties promote healing by reducing reactive oxygen species (ROS), lowering oxidative stress in the wound environment [48]. The synergistic anti‐inflammatory, proliferative, and antioxidant processes work together to create a favorable microenvironment for tissue regeneration, which contributes to the extensive therapeutic benefits of curcumin in clinical settings.

The considerable variation in curcumin formulations and dosing regimens among the reviewed trials presents a major obstacle to synthesizing results. Interventions included systemic oral capsules and various topical formulations such as creams, gels, and complex nano‐formulations. Due to the poor absorption of curcumin, concentrations at the wound site can vary widely, which can directly affect treatment efficacy, making this variability a critical factor [14, 49]. As a result, conducting a meta‐analysis or direct comparison of results is challenging in the absence of established protocols. Moreover, since the effect of curcumin is dose‐dependent, there is a risk of either subtherapeutic effects or, in rare instances of high concentration, possible adverse effects [50, 51].

Despite the mainly good outcomes, it is essential to examine the trials that found no statistically significant therapeutic benefit. For example, Chainani‐Wu et al. [25] discontinued their trial on oral lichen planus after an interim analysis revealed no difference between oral curcuminoids and placebo. The authors observed that a significant, long‐lasting effect from a first round of prednisone given to all patients could have hidden any potential advantage of curcumin. Similarly, Nikpour et al. [33] designed a study to compare the effect of curcumin cream and honey on the healing of episiotomy wounds, reporting no statistically significant difference in wound healing or pain relief between curcumin cream, honey, and the placebo group. These null results do not necessarily rule out the usefulness of curcumin but rather highlight the complexities of clinical study design. Confounding factors, such as the adequate standard of care, co‐interventions, insufficient dosage, or a strong placebo response, can make it difficult to distinguish the therapeutic effect of the substance under investigation.

Curcumin has a consistent and robust safety profile, a key finding presented in this study. Eighty‐four percent of the reviewed articles reported no adverse effects, supported by extensive scientific research. Curcumin has been recognized as “Generally Recognized As Safe” (GRAS) by regulatory authorities such as the United States Food and Drug Administration, based on its long history of use and extensive research [52]. The mild, temporary side effects noted in our review, including mild gastrointestinal discomfort with oral administration and localized burning sensations with topical application, align with clinical trials where high dosages of up to 12 g/day were tolerated well [53]. The strong safety profile is partly due to curcumin's low systemic bioavailability, as it is poorly absorbed, rapidly metabolized, and quickly eliminated, reducing the risk of systemic toxicity [54].

The main strength of this study is its rigorous and transparent methodology. We followed the PRISMA‐ScR criteria and employed a comprehensive search strategy across multiple databases to provide a broad overview of the available clinical evidence. However, some limitations were encountered. Specifically, we were unable to perform a risk‐of‐bias or quality assessment of the studies included. Additionally, the diversity of formulations, treatment durations, and clinical outcomes prevented us from conducting a quantitative meta‐analysis. The exclusion of non‐English publications, along with the potential for publication bias, may have further limited the scope of our findings.

## 5 Conclusions

This extensive scoping study highlights the safety and effectiveness of curcumin as an adjunctive therapy for enhancing wound healing in various contexts. The available clinical evidence consistently demonstrates beneficial outcomes, including faster healing and reduced inflammation, supported by an excellent safety profile with minimal side effects. However, a major issue identified is the considerable variation among the trials examined, particularly regarding curcumin formulations, dosages, and outcome measures. This variability reduces our ability to establish consistent, evidence‐based clinical guidelines. Future studies should focus on large‐scale, standardized trials, systematic reviews along with meta‐analyses, and network meta‐analyses studies to better define the role of curcumin in wound care.

## Author Contributions

M.M.P., N.F.H., designed the study. M.M.P., N.F.H., A.A., Y.D., M.A.S. collected the data. M.M.P., A.A., Y.D., M.A.S. drafted the manuscript. M.M.P., N.F.H., Y.D., M.A.S. finalized the manuscript. All authors reviewed the manuscript and approved the final version. They take full responsibility for the content and writing of this article.

## Funding

This research received financial support from the Vice‐Chancellor of Research at Shiraz University of Medical Sciences under Grant Agreement Number 32392. The authors have no relevant financial or non‐financial interests to disclose.

## Ethics Statement

This study was approved by the Research Ethics Committee of Shiraz University of Medical Sciences (Ethics Code: IR.SUMS.MED.REC.1404.036).

## Conflicts of Interest

The authors declare no conflicts of interest.

## Data Availability

The data that support the findings of this study are available on request from the corresponding author. The data are not publicly available due to privacy or ethical restrictions.
